# Estimation of Peptide Helicity from Circular Dichroism Using the Ensemble Model

**DOI:** 10.1021/acs.jpcb.3c07511

**Published:** 2024-03-12

**Authors:** Uroš Zavrtanik, Jurij Lah, San Hadži

**Affiliations:** Department of Physical Chemistry, Faculty of Chemistry and Chemical Technology, University of Ljubljana, 1000 Ljubljana, Slovenia

## Abstract

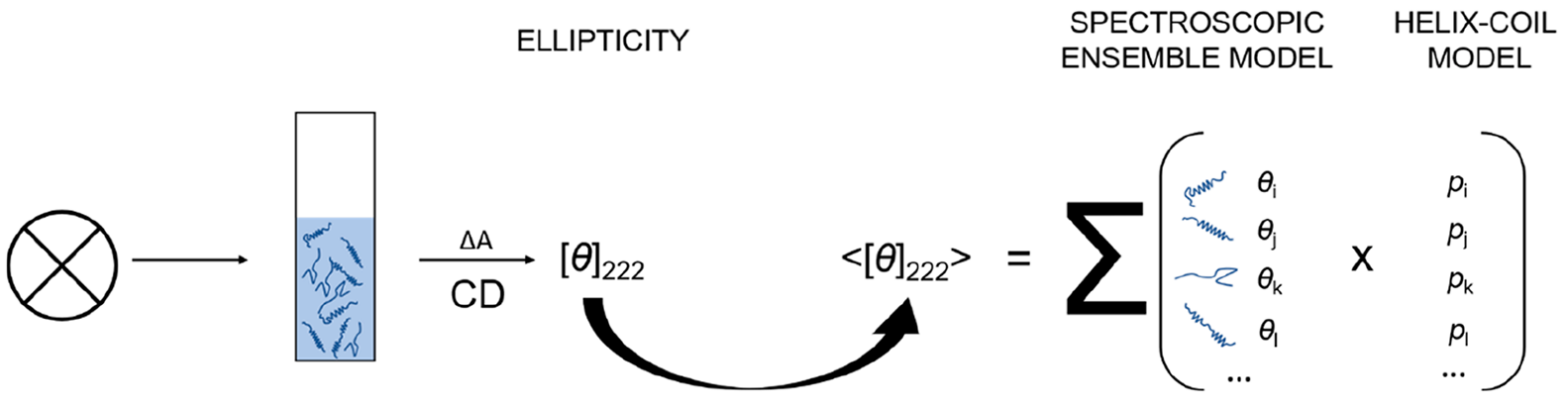

An established method for the quantitation of the helix content in peptides using circular dichroism (CD) relies on the linear spectroscopic model. This model assumes an average value of the helix-length correction for all peptide conformers, irrespective of the length of the helical segment. Here we assess the validity of this approximation and introduce a more physically realistic ensemble-based analysis of the CD signal in which the length correction is assigned specifically to each ensemble conformer. We demonstrate that the linear model underestimates peptide helicity, with the difference depending on the ensemble composition. We developed a computer program that implements the ensemble model to estimate the peptide helicity. Using this model and the CD data set covering a broad range of helicities, we recalibrate CD baseline parameters and redetermine helix–coil parameters for the alanine-rich peptide. We show that the ensemble model leverages small differences in signal between conformers to extract more information from the experimental data, enabling the determination of several poorly defined quantities, such as the nucleation constant and heat capacity change associated with helix folding. Overall, the presented ensemble-based treatment of the CD signal, together with the recalibrated values of the spectroscopic baseline parameters, provides a coherent framework for the analysis of the peptide helix content.

## Introduction

Circular dichroism (CD) is a powerful spectroscopic technique for the quantification of the secondary structure in proteins and peptides, including disordered proteins.^1,2^ For helical peptides, the CD spectrum has two minima at 222 and 208 nm and a maximum at 190 nm, while peptides in the coil conformation have a characteristic negative peak at 200 nm. The minimum at 222 nm is due to the n−π* transition, which is particularly pronounced in α-helix and can be used to quantify the helix content. The transition from helix to coil exhibits an isodichroic point at 203 nm, indicating that a single chromophore (peptide unit) can populate only two states, namely, the helix and coil.^3,4^ For a given peptide conformer with a defined configuration of helix and coil peptide units, the molar ellipticity is the sum of the contributions of all units. In solution, peptides populate an ensemble of *M* different conformers, each with a unique configuration of helical and coil units. For an ensemble of *M* conformers, the measured mean molar ellipticity at 222 nm, [*θ*]_222_, is
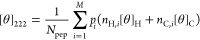
1Here, each *i*-th conformer has *n*_H,*i*_ helix and *n*_C,*i*_ coil peptide units. These contribute [*θ*]_H_ and [*θ*]_C_ to the total CD signal, which correspond to the molar ellipticity of the helical and coil peptide units. The signal contribution of each i-th conformer is weighted by its ensemble probability *p*_*i*_ so that the total signal [*θ*]_222_ is a sum of the probability-weighted contributions of all *M* conformers in the ensemble. To determine the ensemble composition (probability of each conformer) from the measured [*θ*]_222_, one therefore requires values of the helix and coil molar ellipticity ([*θ*]_H_ and [*θ*]_C_), commonly referred to as helix and coil baselines.

The coil baseline has been estimated experimentally using short model peptides where all units were assumed to adopt the coil conformation and the reported [*θ*]_C_ ranges between 600 and 2,200 deg cm^2^ dmol^–1^ at 0 °C^5,6^ (all values in the manuscript are per mole of peptide unit). The estimation of the helix baseline [*θ*]_H_ is more difficult, and its values are still debated.^7^ This is mainly because the [*θ*]_H_ value depends on the length of the helical segment to which it belongs.^8,9^ The so-called helix-length correction is given as
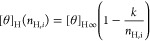
2Here, [*θ*]_H_ (*n*_H,*i*_) is the molar ellipticity of a peptide unit inside the helix segment with *n*_H,*i*_ helical units. [*θ*]_H∞_ is the molar ellipticity of an indefinite helix, and the right-hand term is the correction for the end-effect scaled by parameter *k*. For a sufficiently long helix (*n*_H,*i*_ ≫ *k*), the correction becomes insignificant and [*θ*]_H_ ≈ [*θ*]_H∞_. However, for shorter helical segments, the signal intensity per peptide decreases nonlinearly with the length of the helix segment *n*_H_. Several theoretical and experimental approaches have been used to estimate the helix baseline parameters, but there is no consensus on these values. The proposed values range from −35,000 to −44,000 deg cm^2^ dmol^–1^ for [*θ*]_H∞_ and from 2 to 4 for *k*, but values of up to *k* = 6 have also been proposed.^10^

**Figure 1:**
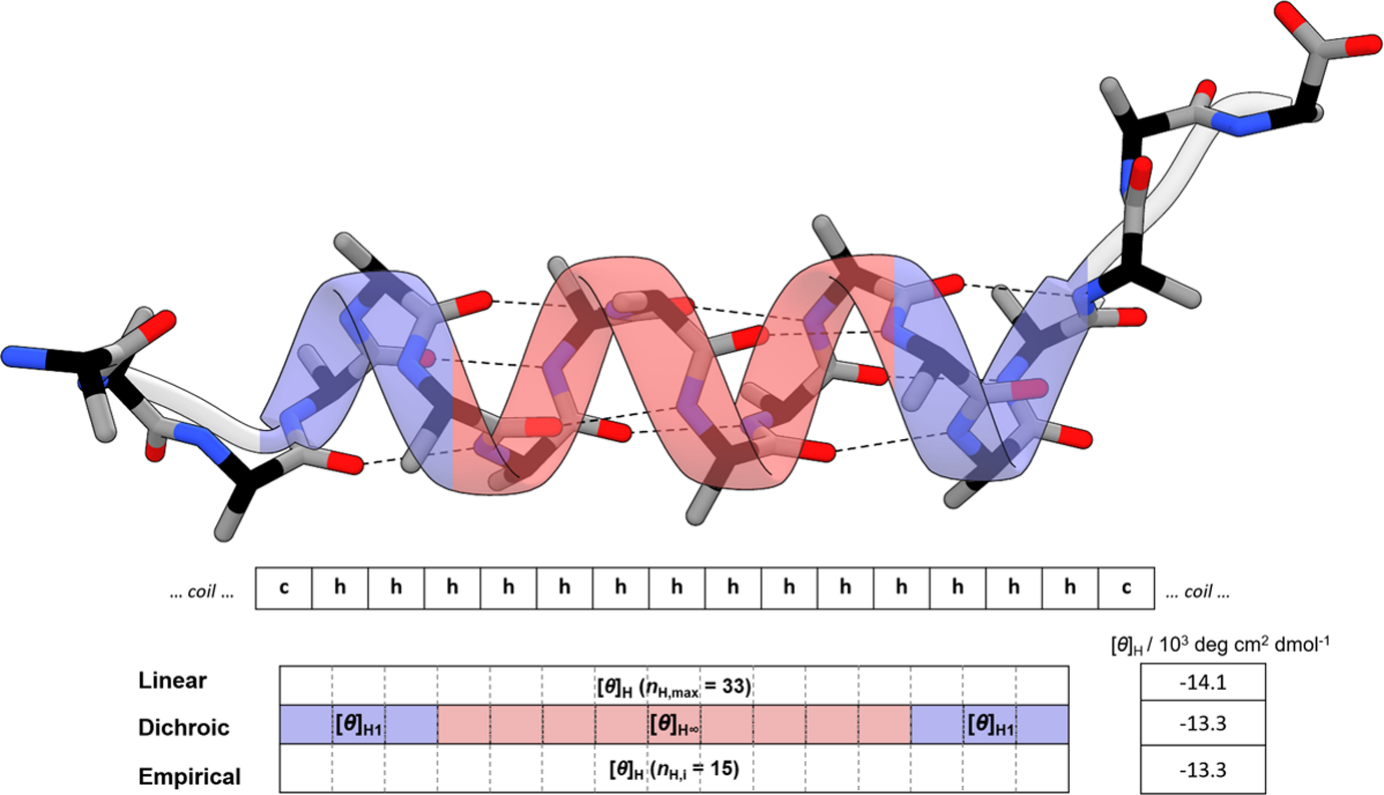
Comparison of the helix-length corrections used by different spectroscopic models. The upper panel shows a peptide conformer with a helix segment of *n*_H_ = 15 helical units out of a total of *N*_pep_ = 33 units. Coil units are shaded gray, single-hydrogen-bonded helical units are in blue, while double-bonded units are shown in red. Only a few units neighboring the helix segment are shown for the sake of clarity. Backbone hydrogen bonds are shown as black dashed lines. The table below shows the corresponding residue definitions (coil or helix conformation) and the helical ellipticity contributions assigned by the linear, dichroic, and empirical length correction. The final helical ellipticity values for this conformer as calculated using different models are listed on the right assuming parameters [*θ*]_H∞_ = −40,000 deg cm^2^ dmol^–1^, *k* = 4, and *N*_pep_ = 33 and eqs 2 and 3.

What is the molecular origin behind the length dependence of the helix CD signal? Kauzmann and Eyring suggested that helical peptide units exhibit unequal restriction of the peptide plane due to the differences in hydrogen bonding in the helix.^11^ The three peptide units at the ends of the helix have a single hydrogen bond, whereas the units inside the helix are double bonded to the *i* – 3 and *i* + 3 peptide groups and therefore are more restricted (Figure 1). This idea is implemented in the dichroic spectroscopic model that explicitly assigns two different spectroscopic contributions to the single- and double-bonded peptide units (Figure 1).^12^ Double-bonded units in the helix center correspond to the situation in an infinite helix and are assigned to [*θ*]_H∞_, while the single-bonded units at the helix ends have ellipticity [*θ*]_H1_. This provides a molecular interpretation of the empirical end-effect parameter *k*, which is related to the difference in molar ellipticity contributions in the dichroic helix length correction as^12^
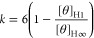
3There is a slight difference between the empirical and dichroic helix length corrections (Figure 1). In the dichroic model, peptide chromophores are counted individually. Therefore, for helical segments with different lengths, the corresponding [*θ*]_H_ will change nonlinearly, up until the helix segment is 6 units or shorter, when [*θ*]_H_ changes linearly and each unit contributes [*θ*]_H1_. In the empirical correction, on the other hand, [*θ*]_H_ changes nonlinearly over the whole range of *n*_H_ values, also for short helix segments.

The most common method for the estimation of peptide helicity relies on the linear model, which uses an approximation for the helix length correction. The linear model does not apply specific helix-length corrections to each peptide conformer based on its *n*_H_ but rather assumes a constant value for all conformers in the ensemble regardless of the length of their helical segment. For all helical peptide units, the same value of ellipticity is used, which is the one corresponding to the helical segment with the maximal length [*θ*]_H_(*n*_H,max_) (Figure 1). This assumption greatly simplifies eq 1 since the sum over the probability-weighted number of helix contributions is then equal to the product of [*θ*]_H_(*n*_H,max_) and the average number of helix residues ⟨*n*_H_⟩. This enables the calculation of the peptide helix content directly from [*θ*]_222_ without a knowledge of the ensemble composition (the probability of each conformer). Even though most studies use the linear model to estimate the peptide helix content, the validity of the linear approximation, to our knowledge, has not been investigated in detail. Furthermore, an approach where the calculation of the CD signal would account for the specific length of helices in different peptide conformers would be more accurate. However, a typical problem with the ensemble models is related to the difficulties in applying conformer-specific length corrections (either a dichroic or empirical correction). This is because the standard procedure for the derivation of the partition function yields an expanded matrix product where spectroscopically distinct states are grouped into the same terms. Two previous studies approached this by using an iterative enumeration algorithm for the derivation of the partition function and used an empirical or dichroic length correction.^10,12^ The study by Shalongo and Stellwagen observed significant differences in peptide helicity between the ensemble dichroic model and linear model, but due to different definitions of the chromophore unit in these models, a direct comparison is difficult.^12^ Just recently, another enumeration algorithm was developed for the efficient calculation of the helix–coil partition function.^13^

In this study, we first developed a new matrix-based algorithm to compute the peptide partition function. The expanded matrix product gives individual terms for different helical conformers, which simplifies the application of conformer-specific helix length corrections. We then theoretically investigate the difference in mean peptide helicity calculated with linear or ensemble models and observe that the linear approximation tends to underestimate the mean peptide helicity. We next apply the ensemble model to re-evaluate CD baseline parameters by analyzing a diverse data set of measured ellipticities ranging from highly helical peptides to peptides populating mostly coil confirmation. Global fitting followed by the Bayesian inference of the model parameters using Markov Chain Monte Carlo provided a new estimate of the CD baseline parameters and of helix–coil parameters for alanine peptides. Compared to the model using the linear approximation, the ensemble model exhibits significantly better parameter stability and lower parameter cross-correlations and uncertainties. Therefore, the ensemble model extracts more information from the data, as it leverages subtle spectroscopic differences between the peptide conformers.

## Materials and Methods

### Peptides and Sample Preparation

The peptides were purchased from ChinaPeptides Ltd. and were at least 95% pure. All contained N-terminal acetyl and C-terminal amide modifications. Peptides were dissolved in milli-Q water at about a 1–3 mg/mL concentration and dialyzed against 10 mM phosphate pH 7.0, 1 M NaCl buffer (phosphate buffer). Before each experiment, these stock solutions were centrifuged and the peptide concentration was determined by measuring the absorbance at 275 nm using an extinction coefficient of 1,450 M^–1^ cm^–1^.^14^ Samples were prepared by diluting stock solutions to a concentration of 100 μM and were used in the CD experiments. Due to systematic differences in the CD signal of the (AAKAA)_6_-GY peptide, a 10% concentration correction was assumed.

### Circular Dichroism

Circular dichroism measurements were performed by using a Jasco J-1500 CD spectrophotometer. All of the samples were measured in a 1 mm quartz cuvette (Hellma) in 10 mM phosphate buffer (1 M NaCl, pH 7). Ammonium *d*-camphor-10-sulfonate (Jasco Ltd.) was measured before and after each measurement to calibrate the intensity of the Xe-ultraviolet lamp. A blank measurement (buffer in a quartz cuvette) was subtracted from each measurement. For thermal melts, ellipticity at 222 nm was measured every 1 °C with an integration time of 8 s. Reversibility was checked by comparing spectra before and after denaturation, and all samples showed complete signal reversibility. The raw signal (ellipticity) was converted to the mean molar ellipticity [*θ*]_222_ of the peptide unit according to the following equation
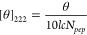
4where *θ* is the measured ellipticity (raw signal) in mdeg, *l* is the optical length in cm, *c* is the peptide molar concentration, and *N*_*pep*_ is the number of peptide units in the peptide. For the blocked peptides (N-terminal acetylation and C-terminal amidation) used in this study, the number of peptide units is different than for the unblocked peptides and equals *N*_*pep*_ = *N*_*res*_ + 1.

### Data Set of Helical Peptides

The literature was searched for suitable coiled-coil systems or short stabilized peptides that remain fully helical over a wide temperature range (high melting temperature). Thermal melting curves were digitized using computer program PlotDigitizer to extract the values [*θ*]_222_ and ∂[*θ*]_222_/∂*T*.^15^ Details of the data points in the data set are summarized in Table S3.

### Lifson-Roig Model

In the LR model, each residue in the peptide chain can be either helical (*h*) or coiled (*c*) based on the geometry of the backbone (Φ, Ψ angles). This results in 2^*N*^ states for a peptide with *N* units. All of these conformers are enumerated by using the matrix method, resulting in an analytical expression for the partition function *Q*. Two parameters determine the partition function: the nucleation parameter *v* and the propagation parameter *w*. The nucleation parameter *v* is assumed to be constant, mainly entropic by origin, and temperature-independent. The helix propagation parameter *w* is related to the formation of a backbone hydrogen bond, and its temperature dependence is expressed by the Gibbs–Helmholtz relation
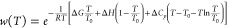
5where thermodynamic parameters (Δ*G*, Δ*H*, and Δ*C*_*p*_) are given at the reference temperature of *T*_0_ = 0 °C, while Δ*C*_*p*_ is assumed to be temperature-independent in the studied temperature range.

The probability for any *i*-th conformer can be calculated by dividing its statistical weight by the partition function *Q* (*v*, *w*) of the whole peptide ensemble.
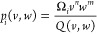
6The conformer contains *n* and *m* units having weights *v* and *w*, while Ω_*i*_ is the total number of possible states with the same composition (degeneracy). Given the probability for each conformer in the ensemble, the CD signal can be calculated using eq 1. More details on the model implementation can be found in the Supporting Information. All calculations were performed using custom Python scripts using numpy and sympy libraries.^16,17^

### Global Fitting and Statistical Analysis

A Bayesian inference analysis of the models was performed using Markov Chain Monte Carlo (MCMC). The analysis was performed using Python probabilistic programming library PyMC.^18^ We adopt a statistical model for the CD data as being generated by the general model function presented in eq 1 with added Gaussian noise (ε)
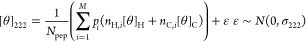
7where σ_222_ expresses the experimental scatter of the measured and acquired CD data. The temperature dependence of helix ellipticity (assuming temperature-independent *k*) as a function of helix length is given by
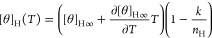
8The temperature dependence of coil ellipticity is given as
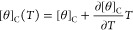
9For all of the model parameters, we set noninformative (uniform) priors that allow model parameters to explore a wide range of values during MCMC.
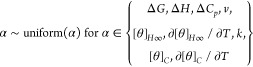
A NUTS (no U-turn) sampler was used to infer posterior distributions of model parameters with 3000 samples (1000 burn-in) (Figure 6C). Correlation coefficients were calculated as Spearman’s rank correlation coefficients.

## Results

### Comparison of Linear, Empirical, and Dichroic Helix Length Corrections

**Figure 2:**
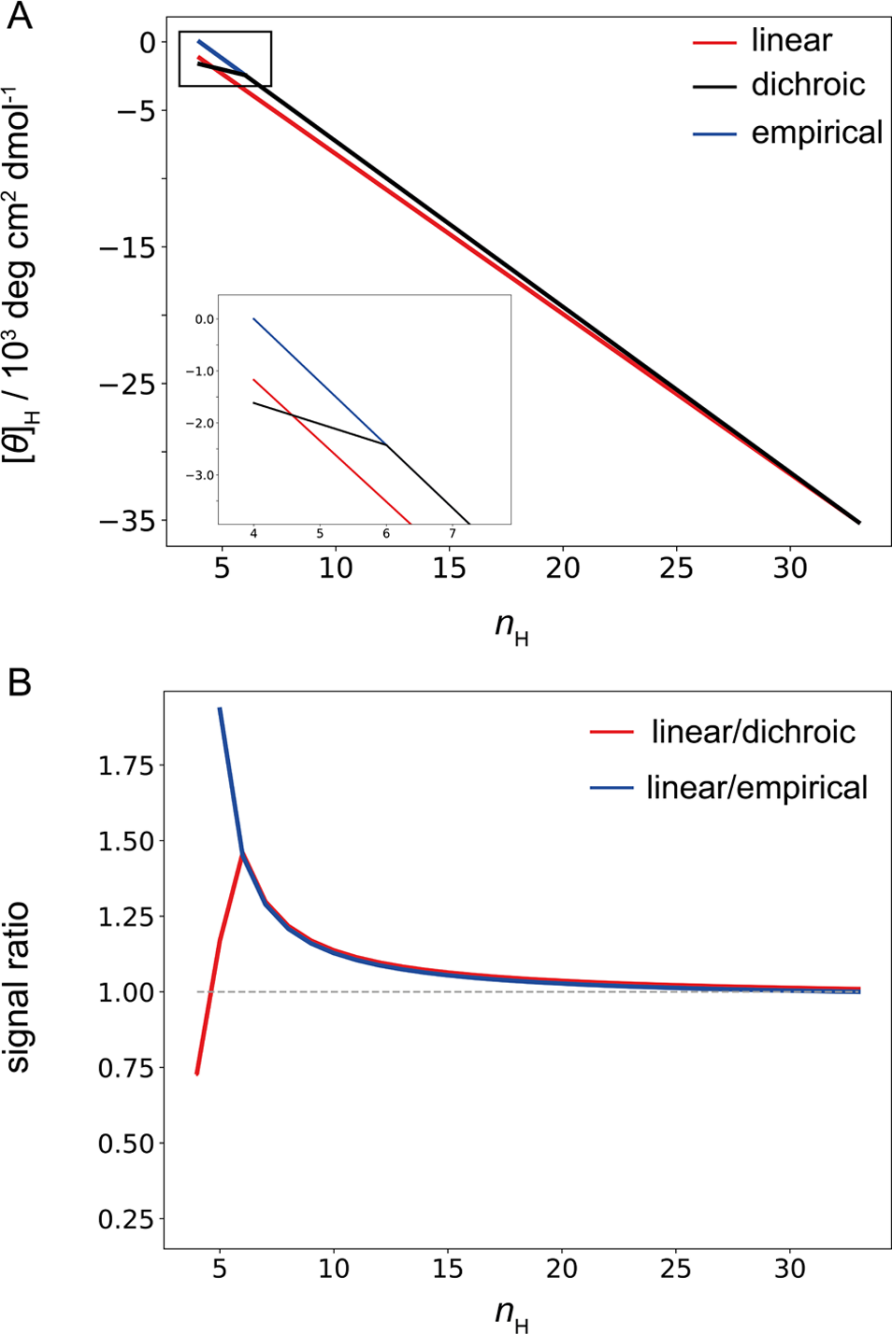
Ellipticity of peptide conformers with increasing length of the helical segment. A) Helix ellipticity ([*θ*]_H_ in units per peptide bond) as a function of the number of helical units (*n*_H_) calculated with different models. Due to the constant length correction approximation, the linear model fails to predict the nonlinear dependence on helix length (red line) given by the two ensemble models, which evaluate the length correction for each conformer individually (black and blue for dichroic and empirical, respectively). The inset shows the ellipticity of short helix segments where dichroic and empirical models diverge. B) The relative difference in the helix ellipticities is calculated in the upper panel. The parameters used in the calculation are the same as in Figure 1.

To understand the differences between the helix-length corrections, we calculated the molar helix ellipticity [*θ*]_H_ for peptide conformers with an increasing number of helical peptide units *n*_H_ using three approaches. With the increasing length of the helix segment (increasing *n*_H_), the ellipticity [*θ*]_H_ increases linearly (becomes more negative, Figure 2A) for the linear model but nonlinearly for the ensemble models with the empirical or dichroic length correction. For the maximal-length helix segment (*n*_H_ = 33), all three models converge to the identical value of [*θ*]_H_ (*n*_H,max_). In the linear model, this is used as a constant per-peptide value for other helical segments of different lengths, while in both ensemble models, [*θ*]_H_ is evaluated for each *n*_H_ individually (eqs 2 and 3). This leads to a nonlinear dependence and an overall lower (less negative) ellipticity (Figure 2A) for ensemble models. The linear model therefore systematically overestimates the CD signal, or for a given signal, it underestimates the true helicity of a conformer. At the level of conformers, this difference is most pronounced for the shorter helices, i.e., by about 1,300 deg cm^2^ dmol^–1^ per peptide (Figure 2A, inset). The ratio of the CD signals estimated with the linear approximation and both ensemble models is shown in Figure 2B and reveals a significant relative difference for short helices (*n*_H_ = 4) and intermediate helices (4 < *n*_H_ < 10), for which the linear approximation leads to an underestimation of 25% and an overestimation of up to 50% of the CD signal, respectively. It should be noted that the magnitudes of all signals and consequently their differences depend on the assumed values of the end-effect parameter *k* (Figure S1). This shows that the linear model gives an “averaged” picture of helix ellipticity and is insensitive to differences among short, intermediate, and long helical segments in conformers.

Ensemble models with dichroic and empirical corrections are nearly identical. The only difference is when the helix segments become short, *n*_H_ ≤ 6 (Figure 2A, inset). In these helices, all peptide units are constrained by only one hydrogen bond, and the dichroic model assigns an identical contribution [*θ*]_H1_ to all units, essentially becoming insensitive to the differences in helix length for *n*_H_ ≤ 6. The empirical model, on the other hand, retains the length dependence even in short helices (eq 2). As shown below, this assumption results in poor fits to experimental data and appears to be incorrect. Another overlooked issue concerns peptide conformers with two or more helical segments. Although such conformers typically constitute only a minor fraction due to unfavorable helix nucleation,^19^ their population increases with the increasing length of the peptide. In the linear model, double-helix conformers are spectroscopically treated as single-helix segments, which again leads to the underestimation of the peptide helicity. On the other hand, the dichroic model explicitly assigns a spectroscopic contribution to each peptide unit and therefore correctly applies an end correction to conformers with multiple helix segments. For conformers with multiple helical segments, the [*θ*]_H_ values estimated with the linear model are overestimated and do not converge even for *n*_H,max_ (Figure S2). Therefore, the linear model overestimates the [*θ*]_H_ of double-helix conformers for the entire *n*_H_ range.

### Implementation of a Matrix Method for the Enumeration of Helical Conformers of Varying Lengths

Although we observe differences in the way the helix-length correction is applied for a given peptide conformer, the relevant question is whether these differences persist at the ensemble level when a probability-weighted mixture of different peptide conformers is present. A traditional and well-established method for enumerating ensemble conformers and calculating their probabilities is based on the Lifson-Roig helix–coil theory implementing matrix formalism.^19^ However, the usual 3 × 3 matrix format gives the joint product terms which contain spectroscopically different states. While this does not represent a problem when the average value of the helix correction is used (as in the linear model), it is not possible to apply the individual per-conformer helix-length corrections as used in the ensemble models. One solution to this problem is the iterative enumeration algorithms that generate every conformer one by one.^10,12^ Here, we use a different approach and develop a modified transfer matrix that gives separate terms for the spectroscopically different states (Materials and Methods and Supporting Information). Briefly, instead of the conventional 3 × 3 matrix, we used an 8 × 8 matrix to separate the helical states in the coil context (*chc* and *chhc* states) from those in the helical context (Table S1). The matrix product is expressed symbolically, with additional statistical weights used as labeling symbols for the *chc* and *chhc* states. These labeling symbols simplify classification of the different terms in the partition function and enable separation of the single- and double-helix segments. Only double-helical segments were considered because the probability of triple or higher helical segments is negligible (<3%) in the studied range of peptide lengths and helix–coil parameters. Overall, the modified matrix provides a simple procedure for the calculation of the length-corrected [*θ*]_H_ contributions for each peptide conformer in the ensemble.

### Linear Model Underestimates the Mean Helicity of the Peptide Ensemble

**Figure 3:**
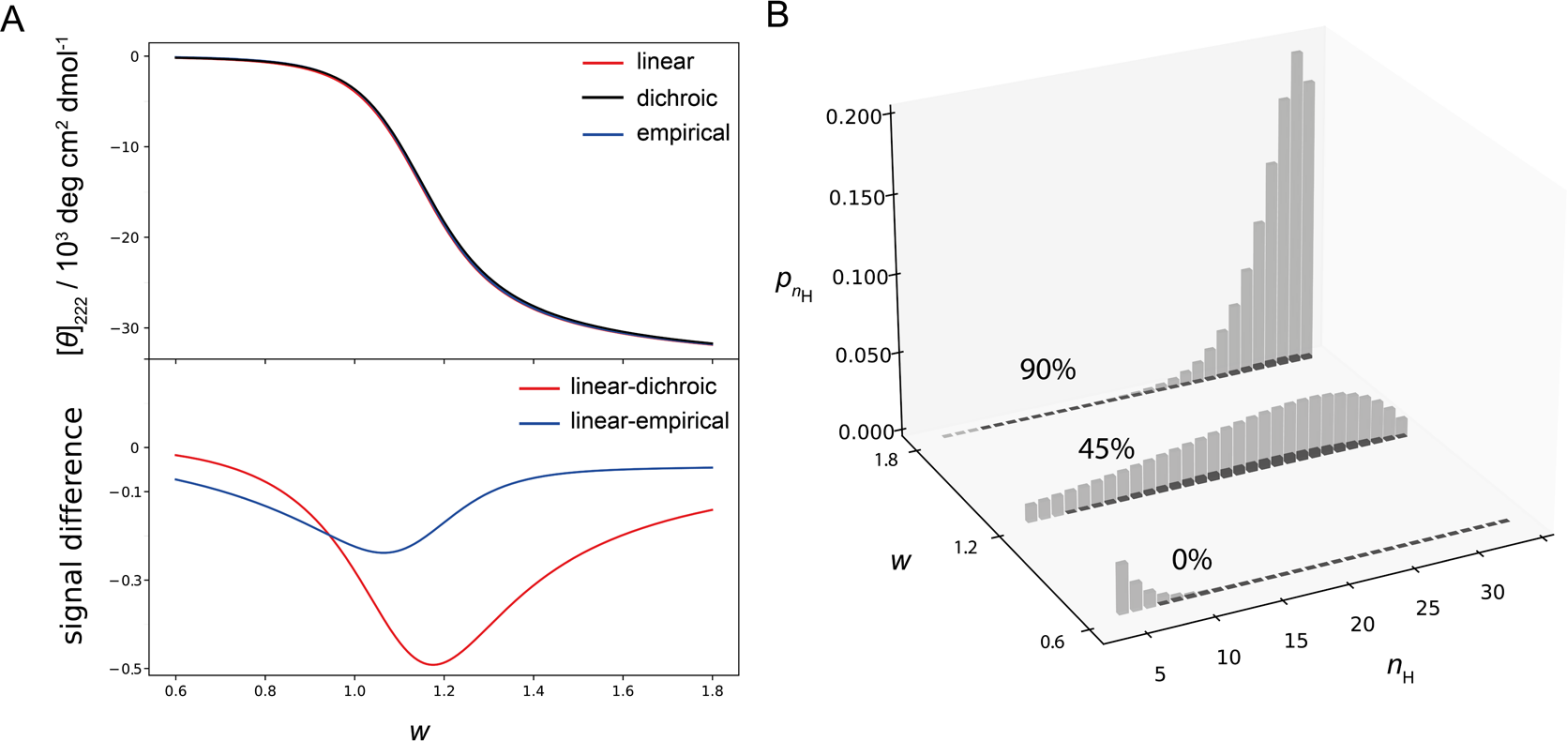
Ellipticity of the peptide ensemble as a function of the propagation constant. A) Changing the propagation constant *w* tunes the overall ensemble helicity. The top panel shows the overall ensemble signal calculated with different models as a function of *w* (*v* = 0.048, other parameters are the same as in Figure 1). The lower panel shows the corresponding signal difference (linear model minus dichroic or empirical). The largest overestimation of the CD signal by the linear model is observed at the intermediate ensemble helicities. B) Ensemble composition (eq 6) for the selected *w* values. The population of peptide conformers with different numbers of helical units (*n*_H_) is shown as bars. Gray bars correspond to conformers with a single helical segment, and darker bars correspond to conformers with two helix segments. Conformers that contribute to the coil (*n*_H_ < 4) are not shown.

Next, we investigate how different spectroscopic models compare at the level of the peptide ensemble. In the Lifson-Roig helix–coil model, the probability of each conformer is calculated using two statistical weights *w* and *v* based on the configuration of peptide units. The propagation constant *w* reflects the favorability of the addition of a helical peptide unit to the existing helical segment, while nucleation constant *v* reflects the unfavorable process of initiating a helix from the coil segment. The values of *w* and *v* define the probabilities of all conformers and therefore the composition of the peptide ensemble. By varying the *w* parameter and keeping *v* fixed, we generate peptide ensembles with increasing mean ensemble helicity ranging from mostly coil ensembles (helix fraction ≈ 0% for *w* = 0.6) to helical ensembles (helix content ≈ 90% for *w* = 1.8) (Figure 3). For each of these ensembles, we calculate [*θ*]_222_ with the linear model and with the ensemble models employing either a dichroic or empirical length correction. All three models predict similar [*θ*]_222_ values as a function of *w* (Figure 3A) that converge to an identical value for the highly helical ensembles, in agreement with previous analysis at the conformer level. The difference in the overall signals (Figure 3A, bottom) shows that the linear model overestimates the total ensemble signal, with the difference peaking when ensembles have intermediate helicity (helix content ≈ 45%, *w ≈* 1.2). The [*θ*]_222_ values differ by up to 5% between the linear model and the ensemble model with the dichroic length correction. We observe that at *w* = 1.2 the ensemble mostly consists of the intermediately sized helix segments (Figure 3B). Although we observed that the signal differences are the highest for the small helices (Figure 2B), these conformers make only a small absolute contribution to the overall [*θ*]_222_. While the relative difference in [*θ*]_222_ decreases with the increase in the helix segment (Figure 2B), the absolute signal contribution increases, explaining the peak at the intermediate helicities. In other words, there is a trade-off between the relative signal difference and the absolute signal intensity, which explains why there is a maximal difference at the intermediate helicity.

**Figure 4:**
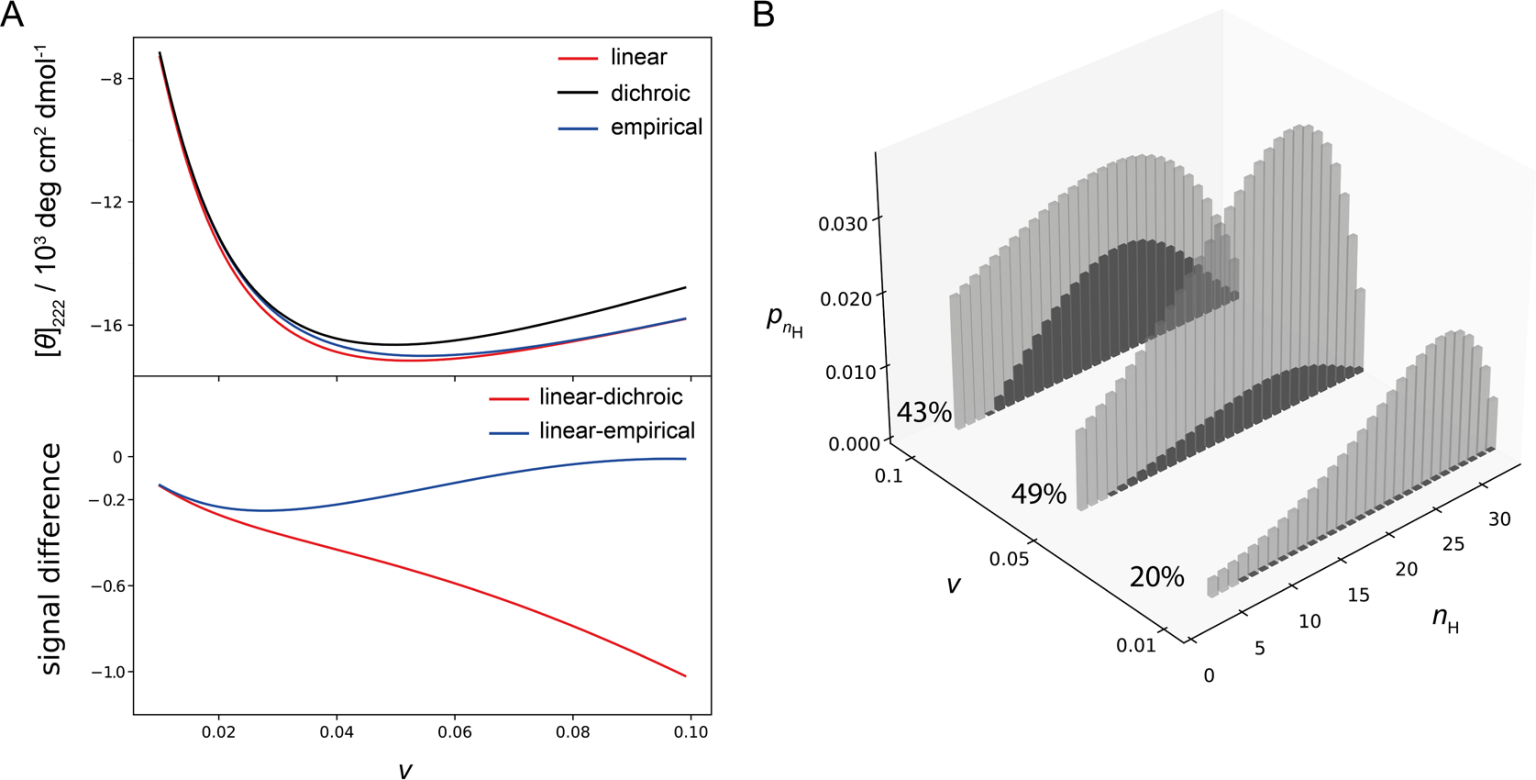
Ensemble ellipticity as a function of the nucleation constant. A) Changing the nucleation constant *v* changes the ratio between single- and double-helix conformers. Top panel shows the total ensemble signal calculated with different models as a function of *v* (*w* = 1.2, other parameters are the same as in Figure 1). Lower panel shows the corresponding signal difference (linear model minus dichroic or empirical). Increasing fraction of double-helix conformers leads to larger overestimate of CD signal by the linear models (red line). B) Ensemble composition (eq 6 in Methods) for the selected *v* values. Population of peptide conformers with different number of helical units (*n*_H_) is shown as bars. Gray bars correspond to conformers with a single helical segment, and darker bars correspond to conformers with two helix segments. Conformers that contribute to coil (*n*_H_ < 4) are not shown.

To evaluate the difference arising from the neglect of the double-helix conformers, we generate another set of ensembles by varying the *v* constant and keeping the *w* fixed. As *v* changes from 0.01 to 0.05, the ensemble helicity increases and all three models give similar ellipticity (Figure 4A). A further increase from 0.05 to 0.1 does not increase the ensemble helicity but promotes the breaking of single-helix conformers into double-helix conformers due to less unfavorable helix nucleation (Figure 4B). The calculated ellipticities now start to diverge (Figure 4A, bottom) due to an increase in conformers with two helix segments. These conformers are not specifically accounted for in the linear model, which applies the helix length correction only once. This is the second reason for the overestimation of the CD signal in the linear model, which is pronounced when ensembles contain a significant fraction of double-helix conformers (Figure 4A).

In summary, the approximations used in the linear model lead to the overestimation of the CD signals and consequently to the underestimation of the actual helix content. This is because the linear model does not apply length corrections to individual conformers in the ensemble and because conformers with multiple helical segments are treated as single-helix conformers. The difference in signal depends on the ensemble composition and the spectroscopic parameter *k* and appears to reach around 5% overall.

### Re-evaluation of CD Baseline Parameters Using the Ensemble Model

The determination of the mean peptide helicity from CD experiments relies on the reliable estimate of CD baseline parameters. The molar ellipticities of the coil and helix are generally considered to be temperature-dependent (eqs 8 and 9 in Materials and Methods), leading to a total of five baseline parameters: [*θ*]_C_, [*θ*]_H∞_, ∂[*θ*]_H∞_/∂*T*, ∂[*θ*]_C_/∂*T*, and the end-effect parameter *k* (eq 2), which we assume is temperature-independent. For the dichroic model, [*θ*]_H1_ and its temperature dependence follow from eq 3. These five spectroscopic parameters define the CD signal of a peptide ensemble at a given temperature. The ensemble composition at a given temperature is defined by four helix–coil parameters: *v*, Δ*G*, Δ*H*, and Δ*C*_*p*_,which define temperature dependence of helix propagation parameter *w* (eq 5). The nucleation constant *v* is considered to be independent of temperature.^20^ Evidently, many parameters are required to describe the variation of helical ensembles monitored by CD, particularly because of the large number of spectroscopic parameters. Previously, the spectroscopic parameters were determined from complementary experiments and individual fits or were adjusted on a case-by-case basis, as discussed further below.

Here, we use a different strategy to estimate spectroscopic parameters. Rather than considering the determination of helix–coil and the spectroscopic parameters as two separate problems, we attempt to determine all parameters by global fitting of the CD data set covering a broad range of temperatures and ensemble compositions. The major part of the experimental CD data consists of the thermal melts of alanine-rich peptides with the sequence (AAKAA)_*n*_-GY, *n* = 3, 4, 5, 6. Alanine residues have a strong propensity for helical structure, while charged lysine residues ensure peptide solubility. Experiments at different peptide concentrations show that peptides are partially helical and monomeric in solution (Figure S3). The residues predominantly sample only the helix and coil but not other conformations, as evidenced by a strong increase in the helix content upon addition of 50% (v/v) trifluoroethanol (Figure S4); therefore, (AAKAA)_*n*_-GY peptides can be considered to be a good system for the application of the helix–coil model, as shown also in previous studies.^21^ However, even in 50% trifluoroethanol, peptides are not fully helical, precluding a precise determination of the helix baseline (Figure S4).

**Figure 5:**
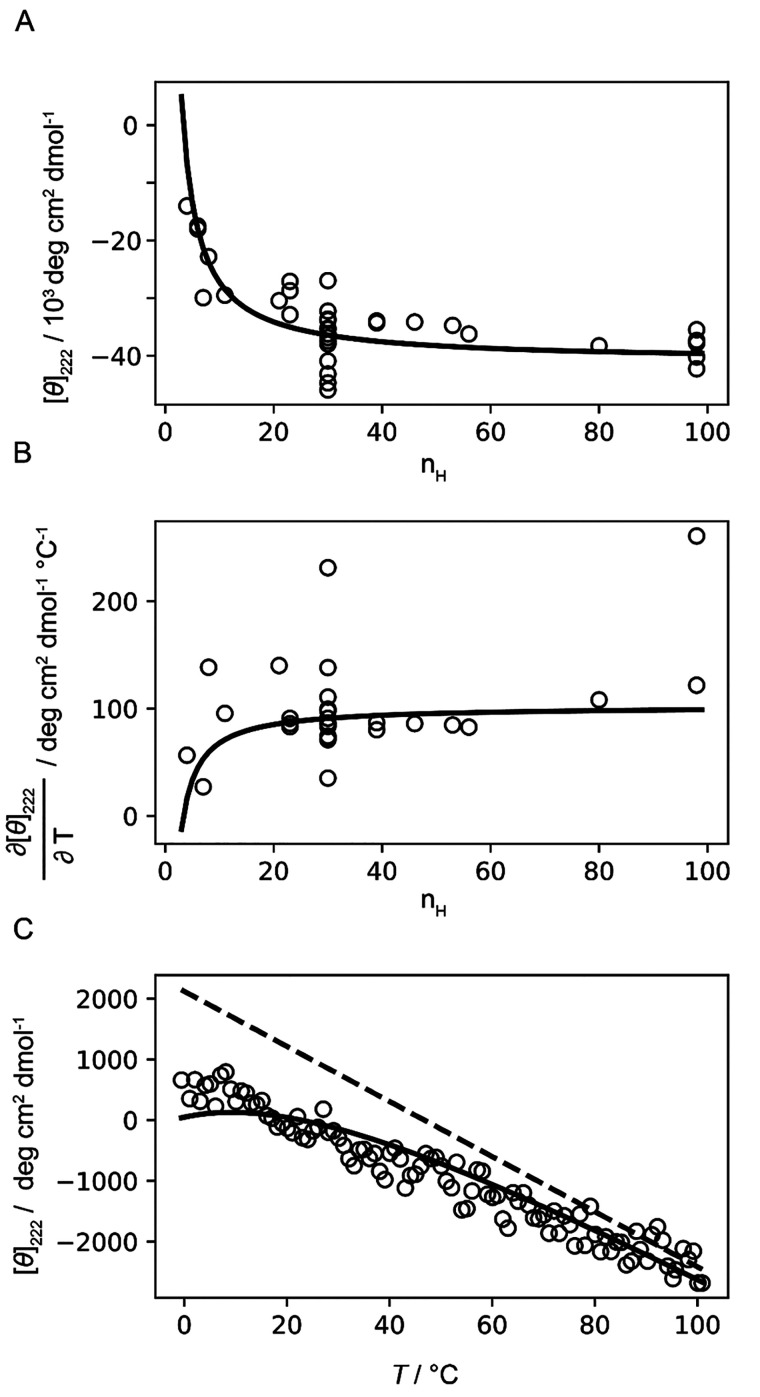
Estimation of helix and coil spectroscopic baselines. A) Symbols show the experimental ellipticity [*θ*]_222_ for peptides where all units are in the helix state at 0 °C. (Further details of these systems are reported in Table S3.) The solid line shows the helix baseline length dependence based on the best-fit values [*θ*]_H∞_ and *k* obtained from the global fit using the dichroic model (eq 2). B) Symbols show the pretransition (*T* ≪ *T*_m_) slopes of measured ellipticities obtained from the thermal melts of helical peptides (Table S3). The solid line shows the helix baseline temperature dependence based on the best-fit values of ∂[*θ*]_*H*∞_/∂*T* and temperature*-*independent *k* (eq 8). C) Symbols show the measured ellipticity for the AAKAA peptide as a function of temperature. The solid line shows the fit to the data using the helix–coil model, which accounts for the minor fraction of helix conformers, while the dashed line shows the “true” coil baseline (where all peptide units populate only the coil conformation) with best-fit values of [*θ*]_C_ and ∂[*θ*]_C_/∂*T* (eq 9).

We therefore assembled [*θ*]_222_ data from the literature focusing on the fully helical peptides of different lengths (Table S3). In these systems, such as coiled coils and chemically stabilized helical peptides,^22^ structural or other evidence suggest that all peptide units are in a helical conformation; therefore, the measured [*θ*]_222_ corresponds directly to [*θ*]_H_(*n*_H,max_). The resulting data show that [*θ*]_H_ decreases nonlinearly with helix length (Figure 5A), enabling the determination of [*θ*]_H∞_ and *k* spectroscopic parameters using global data analysis (the solid line shows the global fit using eq 2 with the ensemble dichroic model). In addition, for the fully helical systems with high thermal stability we determine the change in [*θ*]_222_ with temperature (pre-transition baseline) (Table S3). These measurements are more conveniently expressed as the signal temperature dependence ∂[*θ*]_222_/∂*T*, similar to that mentioned above, for the fully helical systems corresponding to the helix ellipticity temperature dependence ∂[*θ*]_H_(*n*_H,max_)/∂*T* (Figure 5B). The data show considerable scatter but nevertheless enable the determination of the temperature dependence of the infinite helix ellipticity ∂[*θ*]_H∞_/∂*T* (with the solid line showing the global fit using the temperature-dependent term in eq 8 with the ensemble dichroic model). While [*θ*]_H∞_ and ∂[*θ*]_H∞_/∂*T* can be directly extrapolated from data for the peptides with large *n*_H_, the curvature defines the value of the end-effect parameter *k* (Figure 5A, 5B).

To constrain the coil baseline parameters, we measured the CD thermal melting for the short peptide (AAKAA)_*n*_-GY, *n* = 1 (abbreviated as AAKAA, Figure 5C). Previously, short peptides were used to determine the coil baseline, and such measurements were considered to directly correspond to the ellipticity of the pure coil state ([*θ*]_222_ ≈ [*θ*]_C_). This may not be entirely correct, as even short peptides could contain a minor fraction of helical conformers which would bias the baseline determination due to the intense signal of helical conformers. For this reason, we consider the AAKAA data in the global fit and do not impose an assumption of a pure coil state for AAKAA. Indeed, the global fitting with the dichroic model confirms the presence of a minor fraction (about 5%) of helical conformers in the AAKAA peptide ensemble at low temperatures (Figure 5C, solid line) while enabling the determination of the true coil baseline (Figure 5C; the dashed line shows the global fit using eq 9). Due to the minor presence of the helix fraction in AAKAA, the coil baseline lies above [*θ*]_222_ and converges to the data points at high temperatures.

### Re-evaluation of Helix–Coil Model Parameters for the Alanine Peptide Using the Ensemble Model

**Figure 6:**
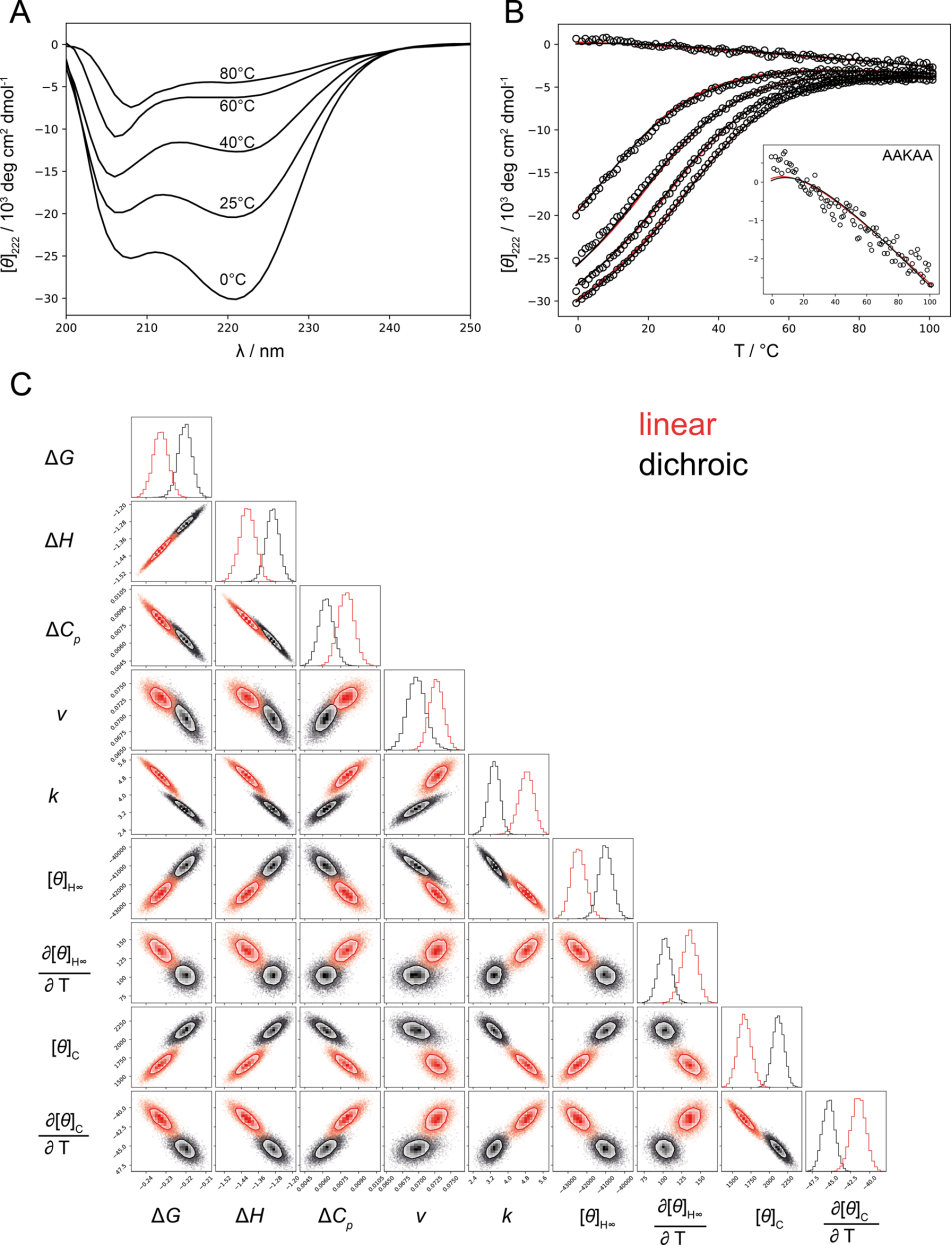
Estimation of spectroscopic and helix–coil parameters by global fitting of CD datasets. A) CD thermal denaturation spectra of the (AAKAA)_6_-GY peptide. Spectra of other peptides in the series are shown in the Supporting Information (Figure S5). B) Global fit to (AAKAA)_*n*_-GY thermal melts with data shown as symbols. The black and red lines represent the global fit using the dichroic and linear models, respectively. The inset shows a close view of the AAKAA data and the model fits. C) Distributions of model parameters and their pairwise correlations for the ensemble dichroic model (black) and linear model (red) obtained using the Markov Chain Monte Carlo method. The parameter’s posterior distributions are shown along the matrix diagonal, with numerical values reported in Table 1.

The ultimate goal of deciphering the relationship between the ensemble composition and the measured CD signal is to obtain a more precise estimate of the helix–coil parameters. We perform a global fit to all CD data simultaneously to re-determine the CD baseline and helix–coil parameters (Figures 5 and 6). The short-peptide and fully helical peptide data set (Figure 5) mainly constrain the spectroscopic parameters, while the (AAKAA)_*n*_-GY, *n* = 3, 4, 5, 6 thermal melts (Figure 6A, 6B and Figure S5) hold the information about the helix–coil parameters. Fitting datasets individually or omitting some of the datasets from the global fit diminishes the quality statistics of model parameters (Figure S6), confirming that each dataset contains some orthogonal information underscoring the necessity of using global model analysis. Interestingly, linear and ensemble dichroic models give almost indistinguishable fits to the data (Figure 6B). Both models describe the data almost perfectly, with only a small but notable deviation for the AAKAA peptide at low temperatures (Figure 6B, inset). The ensemble model with empirical correction performs worse, particularly in the high-temperature region, where the model-calculated ellipticity systematically deviates for all peptides (Figure S7). It therefore appears that the spectroscopic contribution of short helices (*n*_H_ ≤ 6), which dominates under these conditions, is not correctly calculated using the empirical length correction applied to the peptide ensemble.

Given the relatively worse performance of the empirical model, we focus on the fits with linear and dichroic models. We employ the Bayesian inference of model parameters using Markov Chain Monte Carlo and find that despite a similar quality of fit the dichroic model provides an overall statistically better estimate of the model parameters based on several metrics (Figure 6C). Both the uncertainty in the fitted parameters and parameter cross-correlations are lower in fit using the dichroic model (Figure S8). Moreover, the parameter set is more robustly determined, while in the linear model parameters drift upon changing the fitting weights of different datasets (Figure S9). This suggests that the dichroic model can leverage small differences in the spectroscopic contributions of the conformers and thus extract more information from the experimental data. The previously observed differences between the two models are reflected in different values of the estimated parameters. In particular, the linear model predicts higher values for the helix–coil parameters (*v*, *w*, Δ*H*), which are compensated for by the higher end-effect parameter *k*. Table 1 reports the parameters from the global fits with the dichroic model, while those obtained with the linear models are given in Table S4. Finally, we note that neither linear nor dichroic models provide a perfect description of experimental data; a systematic discrepancy for the shortest AAKAA peptide at low temperatures is noteworthy. Whether this is related to the inadequacy of the spectroscopic models or the helix–coil model remains to be investigated (Figure 6B, inset).

**Table 1:** Best-Fit Parameters for the Global Fit to CD Data Using a Dichroic Spectroscopic Model^a^

Spectroscopic parameters		Helix–coil parameters	
[*θ*]_H∞_ [deg cm^2^ dmol^–1^]	–41,000 ± 1,400	Δ*G* [kcal mol^–1^]	–0.22 ± 0.01
∂[*θ*]_H∞_/∂*T* [deg cm^2^ dmol^–1^ °C^–1^]	100 ± 30	Δ*H* [kcal mol^–1^]	–1.3 ± 0.1
*k*	3.4 ± 1.0	Δ*C*_*p*_ [kcal mol^–1^ K^–1^]	0.006 ± 0.002
[*θ*]_C_ [deg cm^2^ dmol^–1^]	2,100 ± 300	*v*	0.07 ± 0.01
∂[*θ*]_C_/∂*T* [deg cm^2^ dmol^–1^ °C^–1^]	–45 ± 5		

a Parameter values are reported per peptide unit at 0 °C and correspond to the mean of MCMC-obtained posterior distributions of model parameters along with their corresponding 2σ deviations.

## Discussion

The main goal of this work was to establish a coherent framework for the calculation of peptide helicity from the measured CD signal. This requires both the appropriate application of a helix-length correction and accurate values of baseline parameters. Elucidating the relation between the CD signal and the underlaying peptide ensemble is important for the reliable determination of helix–coil parameters. We first thoroughly evaluate different approaches to helix-length corrections that, to our knowledge, have not previously been compared on the same footing. The linear model uses the helix ellipticity of the maximal-length helix segment and applies this value to all peptide conformers. We show that the linear model underestimates the true ensemble helicity (Figures 2–4) and that the difference in helicity depends on the ensemble composition. Differences are the largest for ensembles containing conformers with intermediately sized helical segments and those with double-helix segments (Figures 3 and 4). For (AAKAA)_*n*_-GY, *n* = 6, the linear model predicts up to a 5% lower helix content than the dichroic ensemble model (Figure S10). Nevertheless, due to the compensation in model parameters, the linear model can give an excellent fit to the CD data (Figure 6B). However, due to the inherent limitations of the linear model, these parameter values are not as accurate as the ones obtained with the dichroic ensemble model.

The dichroic ensemble model does not use an average value of helix ellipticity for all of the conformers; rather, it assigns a specific value to each conformer which depends on the size of the helix segment. The model is therefore sensitive to the composition of the ensemble, even though the differences in the signals between the conformers are small. Nevertheless, we observe that the dichroic ensemble model can leverage these signal differences to extract more information from the experimental data compared to the linear model. A comparison of the global fits shows that dichroic model parameters have lower uncertainty and cross-correlations and that the parameter set is stable when different weightings of CD data sets are used (Figures S8 and S9). Despite the overall excellent fits, we still observe a small systematic discrepancy between the model-calculated and experimental signals for the AAKAA peptide at low temperatures (*T* < 20 °C). This suggests that some part of the model related to the short helix segments is still inadequate (Figure 6B, inset).

To make calculations with ensemble models more accessible, we developed a new matrix-based method for the calculation of the ensemble-weighted signal contributions. The method is computationally faster and easier to implement compared to the previously used enumeration algorithms.^10,12^ A version of the program that estimates peptide helix content from the measured CD ellipticity implementing the dichroic ensemble model is available on GitHub (Data Availability Statement). While the dichroic model gives more accurate estimates of the peptide helix content, it requires information about the ensemble composition, which needs to be calculated using the provided computer program. A traditional way that does not require the calculation of the ensemble composition is to estimate the helix content using the linear model. This will result in a slight underestimation of the helix content. In such case, we propose using the re-evaluated CD baseline parameters based on the global fit to the CD data set with the linear model (Table S4). With the linear model, the mean helicity (*f*_H_) of a peptide can be calculated directly from the measured mean residue ellipticity (in units of deg cm^2^ dmol^–1^ and at a temperature *T* in °C) using new parameters as
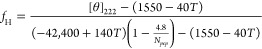
10Note that in order to minimize the discrepancy with the true *f*_H_ values (calculated with the dichroic model) eq 10 contains the spectroscopic parameters from the global fits with the linear model (Table S4). These spectroscopic parameters differ from the physically more accurate values of parameters reported in Table 1 obtained with the dichroic model, but they give a better estimate of the mean peptide helicity when the linear model is used.

There is no clear consensus on the values of the spectroscopic baseline parameters, although they significantly affect the estimation of the peptide helix content and helix–coil parameters. This problem appears to be somewhat obscured in the standard procedure for the analysis of peptide helicity. Typically, the CD signal is first converted to mean helicity using a linear approximation and CD baseline parameters (as in eq 10). Then, the obtained mean helicity is used as the experimental variable and not the actual CD signal. Here we consider the estimation of the spectroscopic and helix–coil parameters as a single problem and start from the raw CD signal given that the mean helicity is essentially also a model-dependent quantity. We assembled a diverse set of CD data and estimated both the spectroscopic and helix–coil parameters using global fitting to the raw CD signal (Figures 5 and 6). Despite a significant number of parameters, we show that they can be determined with sufficient reliability (Figure S6). The value of the coil baseline ([*θ*]_C_ = 2,100 deg cm^2^ dmol^–1^, Table 1) is higher than that reported by Scholtz et al. (640 deg cm^2^ dmol^–1^)^5^ and is close to the value from Luo and Baldwin (2,220 deg cm^2^ dmol^–1^).^23^ In these two studies, short peptides were assumed to represent the pure coil state. In contrast, we do not fix the coil baseline to the AAKAA data; rather, all data support the coil determination via the global fit. Indeed, we observe that even in short peptides a small quantity of helix conformers are present (around 5% at low temperature) and that the true coil baseline lies above the measured data for the AAKAA peptide (dashed line Figure 5C).

The estimated values of the helix baseline agree well with previously published values. The recalibrated value for the ellipticity of the infinite helix is [*θ*]_H∞_ = −41,000 deg cm^2^ dmol^–1^ and that of the end-effect parameter is *k* = 3.4 (Table 1), although the two parameters are partially correlated (Figure 6C). The first estimates of these parameters came from the analysis of reference protein CD spectra ([*θ*]_H∞_ = −39,500 deg cm^2^ dmol^–1^, *k* = 2–4^8^) and theoretical studies ([θ]_H∞_= −45,500 deg cm^2^ dmol^–1^, *k* = 2), while more recent theoretical studies suggest [*θ*]_H*∞*_ = −37,000 and *k* = 2.6–3.^24^ Stabilization of the helical conformation using cosolvents for peptides of different lengths gave [*θ*]_H∞_ = −41,500 and *k* = 3.7, which is in good agreement with our estimate.^10^ Studies which fit the helix–coil models to the CD peptide data often use [*θ*]_H∞_ = −42,500 and *k* = 2.5.^5^ Higher values [*θ*]_H∞_ = −44,000 deg cm^2^ dmol^–1^ and *k* = 3 have been proposed by Luo and Baldwin,^23^ who also revised the ∂[*θ*]_H∞_/∂*T* value from previous +100 to +250 deg cm^2^ dmol^–1^ °C^–1^. However, all studies seem to neglect the length dependence of the helix ellipticity temperature derivate (Figure 5B). Unfortunately, the assembled ∂[θ]_222_/∂*T* data show considerable scatter, obscuring the detailed nature of the helix ellipticity temperature dependence. We therefore considered *k* to be a temperature-independent parameter and find ∂[*θ*]_H∞_/∂*T* = 100 and ∂[*θ*]_H1_/∂*T* = 40 deg cm^2^ dmol^–1^ °C^–1^ (Table 1). When *k* is assumed to be temperature-independent, then this means that the ellipticities of single- and double-bonded helical peptides change synchronously with temperature such that their ratio (eq 3) stays constant at different temperatures and *k* is temperature-independent. It is quite possible that the ratio in ellipticities in eq 3 will change with the temperature, meaning that *k* should be temperature-dependent. Further studies are needed to investigate this. Overall, the helix spectroscopic values reported here largely agree with previous estimates, although there is room for improvement, particularly with respect to the temperature dependence of the helix baseline.

Helix–coil parameters are reported in Table 1 for alanine-rich peptides, which provide an excellent model system for studying the thermodynamic forces governing the folding of the protein backbone. The helix–coil LR model incorporates three fundamental parameters which are closely related to the folding of the protein backbone. The enthalpy change associated with the helix propagation corresponds to the strength of the solvent-exposed main-chain helix hydrogen bond. The obtained value is Δ*H* = −1.3 kcal mol^–1^ and that of the propagation constant is *w* = 1.50 at 0 °C, which both agree with the previous estimates for alanine peptides^5,23,25^ and the statistical analysis of a large set of peptide CD data.^26^ The heat capacity upon helix folding has historically eluded precise determination; however, it provides information on the role of solvation entropy in helix folding.^27^ The estimated heat capacity change is very small, Δ*C*_*p*_ = +6 cal mol^–1^ K^–1^, indicating only a small change in solvation that is expected for the alanine peptide. Although Δ*C*_*p*_ shows a significant cross-correlation with some of the parameters (Figure 6C), the fits are very poor when Δ*C*_*p*_ = 0 is assumed (Figure S11), supporting the existence of a small, non-negative Δ*C*_*p*_. Previous calorimetric studies using DSC and ITC were unable to determine the exact value of Δ*C*_*p*_ and concluded that Δ*C*_*p*_ is zero or very small.^28,29^ Two other studies reported a small negative heat capacity for helix folding of around −4 cal^–1^ mol K^–1^ res^–1^.^30,31^ A study using peptides with the XEARA sequence reported Δ*C*_*p*_ = +8 cal mol^–1^ K^–1^.^32^ This value is similar to ours, although the peptide sequences differ. This perhaps suggests that Δ*C*_*p*_ arises only from backbone interactions; however, the molecular origin of the heat capacity increment in helix folding remains to be investigated. The estimated value of nucleation constant *v* = 0.07 is higher than the previous estimate (*v* = 0.048^33^), indicating that helix initiation is less unfavorable as considered previously. Our estimate appears to be reliable in terms of low cross-correlations and is also supported by a poor fit quality when the usual *v* = 0.048 value is assumed (Figure S12). In most helix–coil studies, the nucleation constant is not being determined but is used as a fixed parameter *v* = 0.048 for the homopolymer model or as *v* = 0.036 in helix–coil models with capping interactions.^34^ These values were obtained from the amide proton exchange experiments.^33^ Our value is closer to that reported by Scholtz et al. obtained as a fitting parameter of the CD thermal melts^5^ and to a more recent analysis using the helix–coil model with an added solvent effect.^35^ The LR model defines the nucleation constant as the ratio of helix and coil conformational integrals in the Φ, Ψ phase space.^20^ Therefore, the value of the nucleation constant is directly related to the change in the backbone conformational entropy, as the peptide adopts a more restricted, helical part of the phase space. The change in backbone conformational entropy upon helix folding appears to be less unfavorable as anticipated based on the traditionally used value *v* = 0.048. This could have important implications for protein folding, as the decrease in the conformational entropy is considered to be the single largest force opposing protein folding.^36^

In conclusion, we show that the commonly used linear approximation of the helix-length correction leads to a small but systematic underestimation of the mean peptide helicity. We develop a model and a computer program that applies a helix-length correction to each peptide conferred in the ensemble based on the corresponding number of peptide units in the helical confirmation. This ensemble model with dichroic length correction is more sensitive compared to the model using linear approximation and enables a reliable determination of the baseline and helix–coil parameters for alanine-rich peptides. Overall, these values could serve as a benchmark for further improvements in helix–coil theory.

## Data Availability

A computer code that implements the dichroic model for the calculation of peptide helix content based on the measured CD ellipticity is available on GitHub at https://github.com/sanhadzi/Dichroic-CD-model.

## Supporting Information Available

Supplementary text on the implementation of the dichroic spectroscopic model and additional experimental and methodological material (PDF)
